# Eosinophilia in Amoxicillin-Induced Rash in Infectious Mononucleosis

**DOI:** 10.7759/cureus.33504

**Published:** 2023-01-08

**Authors:** Ana Abreu, Sofia Nunes, Carmen Botelho

**Affiliations:** 1 Pediatrics, Hospital de Braga, Braga, PRT; 2 Allergy and Immunology, Hospital de Braga, Braga, PRT

**Keywords:** eosinophilia, exanthema, allergy, immunology, epstein barr virus infections

## Abstract

A link between amoxicillin-induced rash in infectious mononucleosis and allergy has been previously reported. However, the pathophysiological cause and aspects are unclear. Additionally, the complex immunological interaction between the host and Epstein-Barr virus needs to be studied.

This article reports a case of amoxicillin-induced rash in infectious mononucleosis resulting in an exuberant rash, facial edema, and marked eosinophilia, which prompted additional workup. Both the eosinophilia and the rash brought to light a possible association with a persistent delayed-type hypersensitivity. Further scientific discussion and investigation can identify predictive indicators that can portend clinical outcome.

## Introduction

From 1968 onward, Epstein-Barr virus (EBV) was first implicated as one of the causative agents of infectious mononucleosis (IM), and an increasing number of skin eruptions in patients erroneously treated with antibiotics was observed. Thereafter, comprehension of this phenomenon has greatly evolved. Nevertheless, various aspects regarding the interaction of the underlying pathophysiological pathways remain unclear [1,2]. Early reports concerning this adverse reaction estimated its incidence to be between 80% and 100%. However, retrospectively, it has been pointed out that insufficient methodology and sample size limitations may have hampered data reliability [1]. More recent studies have stated that the incidence may be much lower than previously reported [3-5].

In the past, skin tests were neither required nor recommended, as skin rashes following aminopenicillin intake in the setting of mononucleosis were considered nonallergic phenomena, arising from a transient loss of immune tolerance. However, a persistent delayed-type hypersensitivity may emerge concomitantly or within IM in some patients, especially in severe cutaneous manifestations, which are usually rare in primary EBV infection [1,5,6]. In fact, there is recent evidence that these allergic reactions to aminopenicillin may be more prevalent than previously assumed and published [5]. This finding may now impact our approach in such situations, warranting additional investigation and follow-up. However, more studies are required in order to identify clinical and laboratory aspects with predictive value to guide our practice.

This article reports a case of amoxicillin-induced rash in IM, highlighting some atypical findings and uncovering some of the gaps in our pathophysiological understanding of this complex host-virus interaction.

## Case presentation

A previously healthy 15-year-old female presented to the pediatric emergency department with a generalized skin eruption, with a rapid cephalocaudal progression, associated with mild pruritus and facial swelling. She was prescribed amoxicillin for six days for the treatment of sore throat associated with persistent halitosis and malaise. The last dose of antibiotic was reportedly taken 12 hours before the onset of the rash. There was no history of fever or any recent drug intake. She denied having stridor, dyspnea, syncope, vomiting, abdominal pain, or other gastrointestinal complaints. She was unaware of any close contact with sick people.

**Figure 1:**
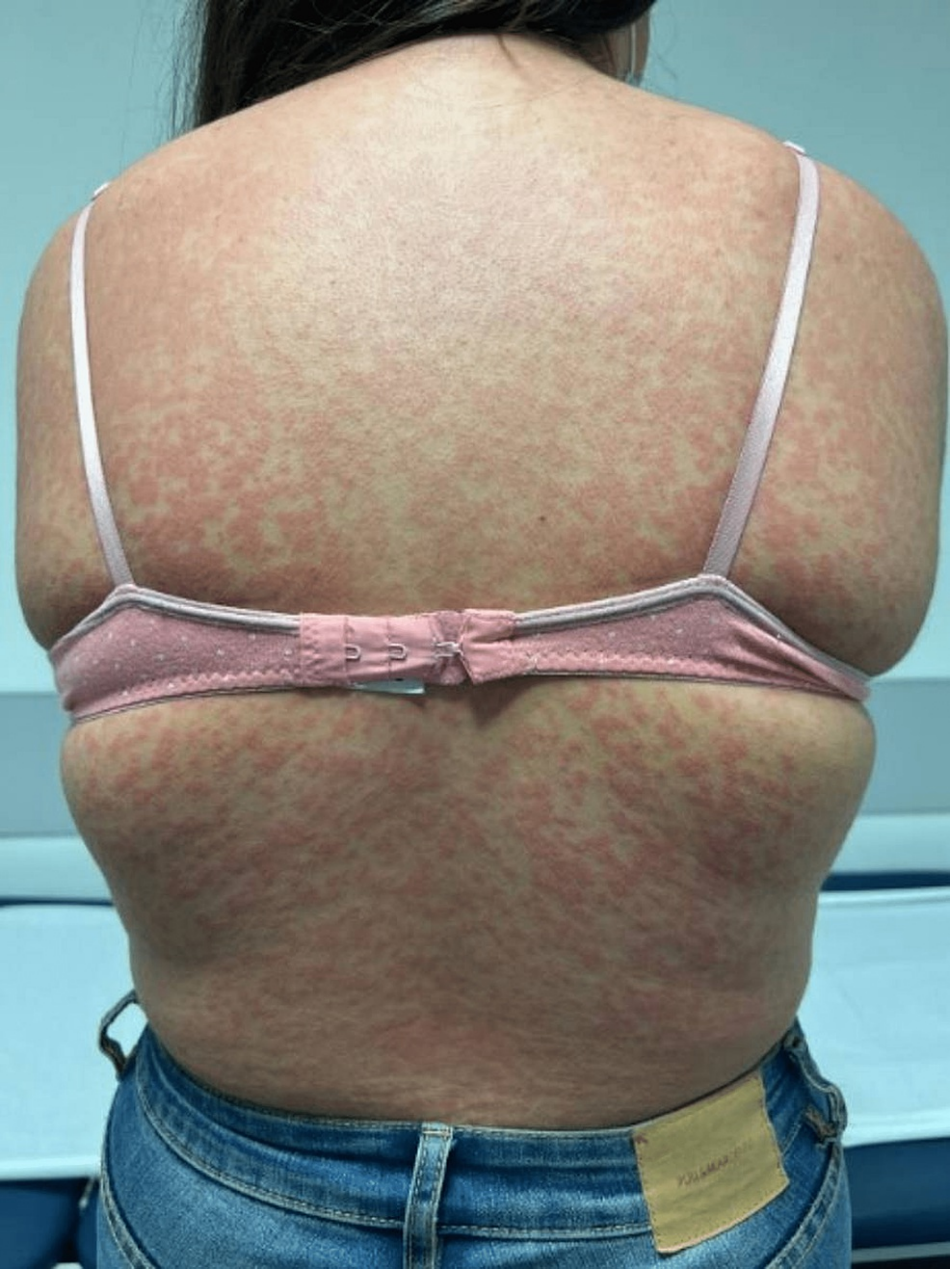
Picture of the patient’s back showing a symmetrically distributed maculopapular exanthem upon hospital admission.

**Figure 2:**
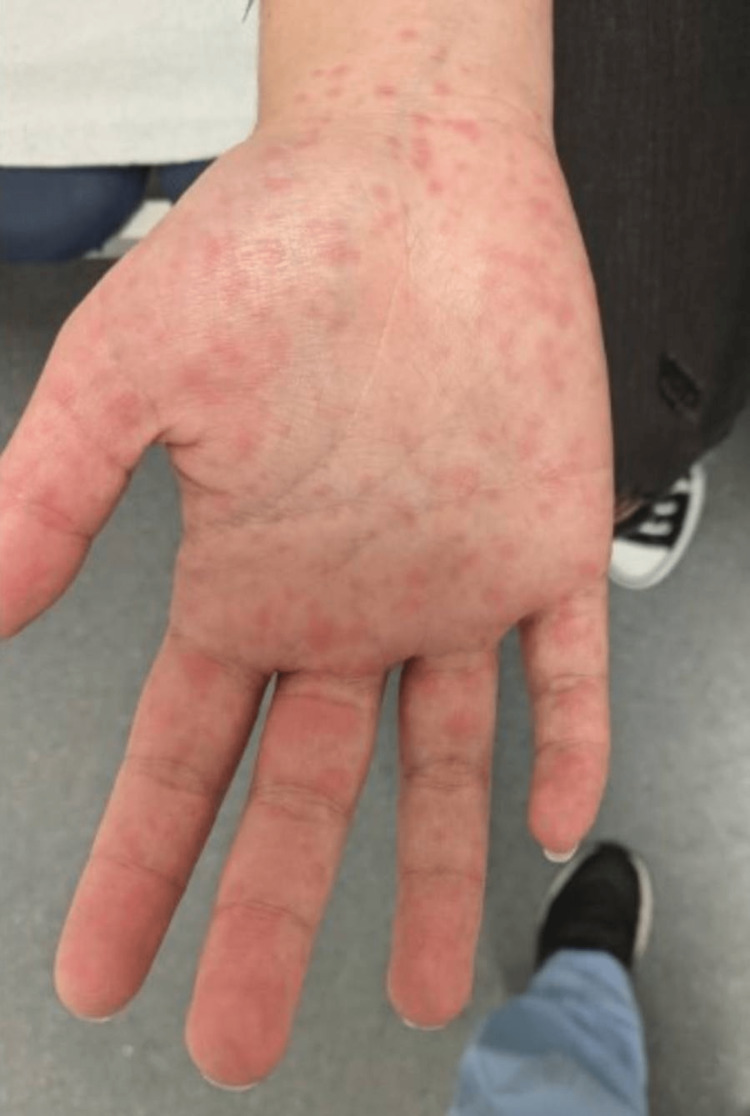
Picture showing the involvement of palms upon hospital admission.

**Figure 3:**
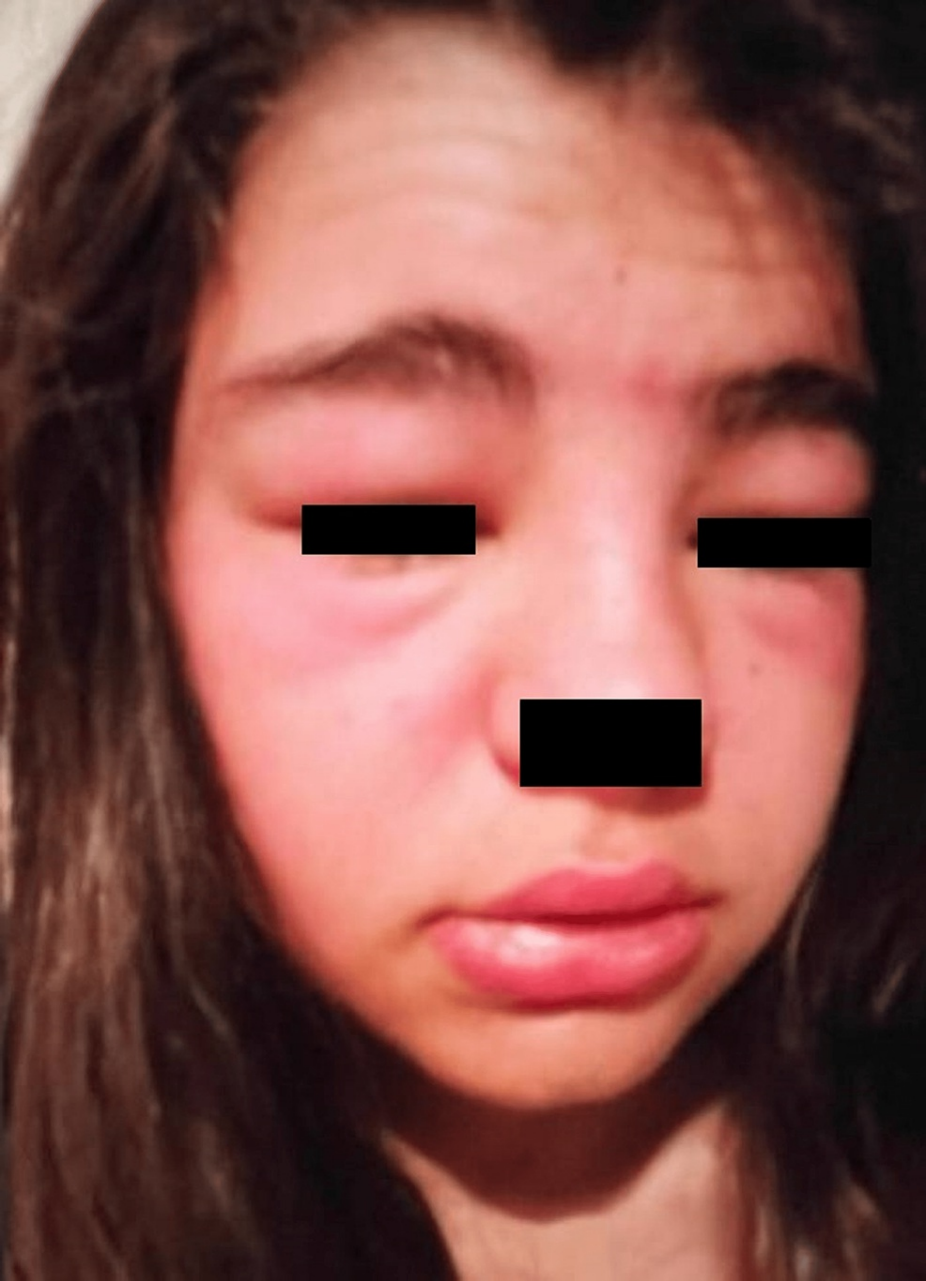
Picture of the patient’s facial swelling upon hospital admission.

On examination, the patient presented with good general condition. She was hemodynamically stable and apyretic, with percutaneous oxygen saturation of 100% and no signs of respiratory distress. There was a generalized symmetrically distributed maculopapular exanthem, more prominent in the trunk (Figure 1), involving the palms and soles (Figure 2), a marked periorbital and facial edema (Figure 3), and erythema of the pharynx. Cervical non-tender, enlarged lymph nodes were palpable, but there was no splenomegaly or hepatomegaly. The remaining physical examination was unremarkable.

Blood tests revealed an elevated atypical lymphocyte count and marked eosinophilia. Aspartate aminotransferase, alanine aminotransferase, and C-reactive protein were mildly elevated. Creatinine level was just above the reference range, with normal blood urea and no ionic imbalances (Table 1). The Paul-Bunnell test was positive.

**Table 1:** Laboratory findings over time. ALT, alanine aminotransferase; AST, aspartate aminotransferase; CRP, C-reactive protein

Laboratory parameters	Upon admission	Day 2	Day 5	Reference range (absolute count when applied)
Leucocytes/uL	11,000	10,600	10,600	4,000-13,500
Lymphocytes/uL (%)	700 (6)	5,000 (47.5)	3,600 (33.7)	1,200-5,000
Atypical lymphocytes/uL (%)	1.100 (10)	-	-	-
Eosinophils/uL (%)	4,300 (39)	900 (8.5)	100 (1.2)	0-500
ALT (U/L)	162	146	95	7-40
AST (U/L)	93	71	30	<35
Urea/creatinine (mg/dL)	30/0.9	34/0.9	35/0.7	19-49/0.46-0.81
CRP (mg/L)	2.7	2.9	0.3	< 0.5

Given the initial findings, further laboratory investigation was carried out. Coagulation tests, troponin, NT-proBNP, myoglobin, and urinalysis showed no abnormalities. Electrocardiogram was normal. Serological assays for EBV favored acute infection (Table 2).

**Table 2:** Epstein-Barr virus serologic results, performed upon admission. EA, early antigen; EBNA, Epstein-Barr nuclear antigen; IgG, immunoglobulin G; IgM, immunoglobulin M; VCA, viral capsid antigen

Laboratory results (U/mL)		Interpretation
Anti-VCA IgM	134	Positive
Anti-VCA IgG	27.7	Positive
Anti-EA IgG	17.8	Doubtful
EBNA IgG	<<3.00	Negative

The patient was first treated with intravenous clemastine 2 mg and a single dose of hydrocortisone 200 mg, with no improvement of the rash or facial edema. Considering her good general condition, she was discharged from the emergency department after a few hours with indication for re-evaluation in the short term (two days). She was instructed to take prednisolone 60 mg orally (1 mg/kg) for three days and desloratadine 5 mg for pruritus control. A rapid normalization of eosinophilia was observed (Table 1), and the exanthema totally resolved by the eighth day after discharge. The patient was then referred for follow-up in an immunoallergology consultation for assessment of an allergic reaction. Initial ambulatory investigation revealed a total and amoxicillin-specific immunoglobulin E within normal reference values.

## Discussion

In the setting of a possible hypersensitivity reaction, peripheral blood eosinophilia often prompts concern as it is classically associated with severe and organ-specific reactions [7]. The most representative example of the aforementioned condition is a drug reaction with eosinophilia and systemic symptoms (DRESS) syndrome, a rare and potentially life-threatening condition that occurs after exposure to sulfonamides, antiepileptics, and antibiotics. Symptoms in the DRESS syndrome begin approximately three to eight weeks after exposure to the offending agent, and the classic presentation includes fever, lymphadenopathy, and extensive skin rash in association with visceral organ involvement, including hepatitis, encephalitis, pneumonitis, hemophagocytic syndrome, and multi-organ failure [2]. As for the cutaneous manifestations, the most characteristic lesions during the early phase are pinhead-sized pustules with blisters occasionally present, mainly limited to the wrists. Mucosal surfaces, along with palms and soles, are usually spared, which is not the case regarding amoxicillin-induced rash in IM, and as seen in our patient [7]. Nevertheless, periorbital and facial edema is considered another hallmark feature of the disease and was notorious in our patient [6]. As for Hoagland sign, an early yet relatively rare sign of IM, the edema is restricted to the upper eyelid. Eosinophilia (usually >700/microL) and atypical lymphocytosis are the main laboratory findings, apart from others that indicate organ involvement [6,7]. Among the multiple factors that interact in its genesis, we find the reactivation of certain herpes family viruses, including EBV [2,6,7]. In fact, various clinical and biological manifestations actually reflect the systemic reaction and immune response against reactivation of human herpes viruses [2]. Therefore, DRESS syndrome and IM, especially if an antibiotic-induced rash is present, share some clinical and analytical features. These include atypical lymphocytosis, hepatocellular lesion with increased aspartate aminotransferase (AST) and alanine aminotransferase (ALT), lymphadenopathies, and occasionally an upper-airway infection-like prodrome, apart from the exanthema [7]. Regarding our case, the presence of marked eosinophilia further overlapped these two clinical entities and required more comprehensive tests for differential diagnosis. A score of 4 according to the Registry of Severe Cutaneous Adverse Reactions (RegiSCAR) classified DRESS as probable. However, the adolescent’s good general condition, absence of fever, timing of symptom onset regarding antibiotic intake, serological results, and overall clinical evolution rendered the amoxicillin-induced rash in IM the most likely diagnosis.

In this case, peripheral eosinophilia may reflect a milder type IVb reaction, which involves a Th2-mediated immune response with secretion of interleukin (IL)-4, IL-13, and IL-5 [8]. Acting as a common pathway between different clinical entities, such as DRESS syndrome, allergic asthma, and, probably, benign drug-induced eosinophilia, IL-5 induces differentiation, activation, and chemotaxis of eosinophils, which often modulate allergic inflammation. Nevertheless, the humoral and cellular mechanisms underlying most cases of drug-related eosinophilia need to be clarified [8]. The authors would like to raise awareness for the atypical characteristics found in a relatively common reaction that should prompt referral, warranting a further allergy workup and allergen avoidance. Further scientific investigation should address the potential risk of allergy and immunologic disorders, as well as predictive factors, thus establishing the most adequate follow-up.

## Conclusions

The amoxicillin-induced rash in IM is still a matter of controversy and may be a prelude to a persistent delayed-type hypersensitivity. The complex interaction between EBV and host may be the common ground for many shared features between DRESS syndrome and amoxicillin-induced rash in IM. However, marked eosinophilia is a distinctive finding in DRESS syndrome. The authors are unaware of other cases of eosinophilia in aminopenicillin-induced rash in IM and question the implications for allergy risk that shaped our patient’s follow-up. Although it can probably be framed in an allergic inflammatory process, the full understanding of the underlying pathophysiological mechanisms may shed some light on this matter and allow recognition of both clinical and laboratory predictive factors. Finally, it is important to underline the need for a more judicious antibiotic prescription, mitigating both antimicrobial resistance and avoidable adverse reactions.
